# Preoperative medical therapy for acromegaly: current knowledge and clinical significance

**DOI:** 10.3389/fendo.2025.1636047

**Published:** 2026-01-02

**Authors:** Dan He, Qinyi Wang, Zhifeng Sheng, Guohua Li

**Affiliations:** 1 Department of Endocrinology and Metabolism, Xiangtan Central Hospital (The Affiliated Hospital of Hunan University), Xiangtan, China; 2 Health Management Center, National Clinical Research Center for Metabolic Diseases, Hunan Provincial Clinical Medicine Research Center for Intelligent Management of Chronic Disease, Hunan Provincial Key Laboratory of Metabolic Bone Diseases, Department of Metabolism and Endocrinology, The Second Xiangya Hospital of Central South University, Changsha, Hunan, China

**Keywords:** acromegaly, growth hormone, preoperative medical therapy, somatostatin analogs, molecular biomarkers

## Abstract

Acromegaly is a chronic endocrine disorder characterized by excessive secretion of growth hormone (GH), predominantly caused by pituitary adenomas. Despite advancements in neurosurgical techniques, the surgical remission rates for invasive macroadenomas or giant adenomas remain unsatisfactory. Therefore, multimodal treatment strategies, including preoperative medical therapy (POMT), have been implemented to improve patient outcomes. Among these, first-generation somatostatin receptor ligands (fg-SRLs) have been the most extensively studied preoperative agents; however, their clinical efficacy in enhancing postoperative remission remains controversial. In recent decades, ongoing research into novel drugs and molecular targets are reshaping the therapeutic landscape of POMT. Beyond traditional clinical models and functional assays, the integration of advanced imaging modalities and molecular biomarkers promises to refine patient stratification, particularly for individuals with suboptimal responses to transsphenoidal surgery (TSS). Furthermore, novel SRL formulations and the identification of new molecular targets could further expand the therapeutic landscape of POMT. In this narrative review, we systematically summarize the latest research advancements in POMT for acromegaly and discusses potential therapeutic strategies and persisting obstacles in this field.

## 1 Introduction

Acromegaly is a rare and insidious disease, typically caused by pituitary tumors that continuously secretes excess growth hormone (GH). This disorder elevates the risk of complications, including left ventricular hypertrophy, hypertension, diabetes, osteoporosis, and sleep apnea, which significantly impair the patient’s quality of life and lifespan (1). Transsphenoidal microsurgery (TSS) is the preferred treatment of choice for most patients with active acromegaly. However, based on the 2000 remission criteria, the recurrence rate of acromegaly in patients treated exclusively with TSS is exceedingly high (2, 3). With advancements in biochemical testing techniques, the 2010 guidelines established more stringent biochemical remission criteria (4). Under these criteria, the surgical remission rate for microadenomas in experienced centers ranges from 56% to 100%, while the remission rate for large adenomas, which constitute 77% of GH-secreting tumors, is only between 27.8% and 61% (5–8). ​In cases of large adenomas or those significantly invading the cavernous sinus (CSI), the cure rate remains relatively low (9). Therefore, medical therapy and radiotherapy have become important adjuncts to surgical treatment. To improve the remission rate of acromegaly, some clinicians have proposed implementing preoperative medical therapy (POMT) in patients with anticipated low rates of TSS remission (10–13). Recent consensus guidance indicates that POMT may be considered in carefully selected patients (for example, when surgical cure is unlikely, surgery will be substantially delayed, or peri-operative risk is high), whereas routine use to enhance postoperative remission is not recommended (14). Advances in imaging, molecular biomarkers, and drug development have deepened the understanding and optimized the management of acromegaly (15). Novel imaging approaches and biomarker studies offer potential for predicting POMT responsiveness, with evidence linking POMT to favorable modulation of markers related to apoptosis and tumor invasiveness (15, 16). This narrative review aims to summarize the new evidence for POMT and outlines future directions to support the development of individualized treatment strategies for acromegaly.

## 2 Literature search strategy

A narrative review of the literature was conducted to summarize the current evidence on POMT for acromegaly. The search was performed using the following databases: PubMed, EMBASE, and Web of Science. Key search terms included “preoperative medical therapy,” “acromegaly,” “somatostatin receptor ligands,” “growth hormone receptor antagonists,” and “dopamine agonists.” The literature search covered studies published up to June 2025. No specific restrictions were applied regarding study design or publication type, but the focus was on studies directly related to the clinical management of acromegaly and the application of POMT.

## 3 The concept of POMT for acromegaly

The exploration of POMT for acromegaly commenced over 40 years ago (17). At that time, only one-third of patients with large adenomas attained surgical remission, and nearly all patients with tumors involving the parasellar region failed to be cured through surgery (17, 18). Meanwhile, researchers suggested preoperative treatment with bromocriptine for prolactinomas, as it inhibits prolactin secretion and induces tumor shrinkage in most prolactin-secreting pituitary tumors (19). This raised the possibility of applying POMT to pituitary GH adenomas with poor surgical outcomes. Barkan AL et al. first evaluated POMT in ten previously untreated patients with invasive large adenomas, demonstrating that preoperative somatostatin receptor ligands (SRLs, e.g., SMS 201-995) markedly reduced GH and IGF-1 levels and resolved or diminished parasellar and cavernous sinus invasion (17). This approach achieved an 80% short-term postoperative remission rate, compared with 31% in a matched surgical-only cohort (17). Subsequent studies have increasingly focused on using POMT to optimize preoperative biochemical control, reduce surgical risks, facilitate complete tumor resection, and improve postoperative remission outcomes (13, 20).

## 4 Research progress on POMT for acromegaly

The pharmacological treatment options for acromegaly can be categorized into three main groups: somatostatin receptor ligands (SRLs), which are recommended as the first-line treatment for patients who are not candidates for surgery; growth hormone receptor (GH) antagonists, which serve as adjunctive therapy for severe cases beyond the first-line treatment; and dopamine agonists (DAs), which are used as adjunctive therapy for mild cases (15, 21, 22). In the field of POMT research, first-generation somatostatin receptor ligands (fg-SRLs) are the most extensively studied, whereas data for other agents are scarce and provide only limited support for their use in treatment strategies (**Table 1**).

**Table 1:** Comparative overview of drugs available for POMT in acromegaly.

Drug class	Representative drugs	Mechanism	Administration route	Key POMT evidence	References
First-generation SRLs	Octreotide LAR; Lanreotide depot	SSTR2 agonism; peripheral inhibition of GH/IGF-1 axis	Intramuscular injection or subcutaneous injection	3–6 mo preop lowers GH/IGF−1; ~50% achieve >20% tumor shrinkage; higher preop doses linked to better long−term remission	(49–55, 84, 85)
Second-generation SRL	Pasireotide LAR	Multi−receptor ligand (SSTR1/2/3/5) with high affinity for SSTR5 and SSTR1; stronger GH/IGF−1 axis suppression vs fg−SRLs	Intramuscular injection	Evidence remains limited; no conclusive benefit on surgical outcomes.	(27–33)
GH receptor antagonist	Pegvisomant	Blocks hepatic GH receptor	Subcutaneous injection	Normalizes IGF−1 and improves metabolic/OSA/arrhythmia outcomes	(34, 38–43)
Dopamine agonist	Cabergoline	D2 receptor agonist; antiproliferative effects	Oral	Improves biochemical control preoperatively; volume change mainly in GH/PRL co−secreting tumors	(44–46)

fg-SRLs, first-generation somatostatin receptor ligands; GH, growth hormone; IGF-1, insulin-like growth factor 1; SSTRs, somatostatin receptor subtypes; LAR, long-acting release.

### 4.1 SRLs

SRLs act by binding to somatostatin receptor subtypes (SSTRs), inhibiting adenylate cyclase, lowering cyclic adenosine monophosphate (cAMP) and intracellular calcium levels, and suppressing GH secretion (23). SSTR2 and SSTR5 mediate antiproliferative effects via tyrosine phosphatase activation and ERK1/2 inhibition, while SSTR2 and SSTR3 contribute to apoptosis (24, 25). Additionally, since hepatocytes also express SSTR2 and SSTR3, SRLs exert peripheral inhibitory effects on the GH-IGF-1 axis (26). Although natural somatostatin can bind to five different SSTRs, SRLs exhibit varying affinities for SSTR1-5. First-generation SRLs, such as octreotide and lanreotide, primarily bind to SSTR2, with a lower affinity for SSTR5 (27). The novel multi-receptor ligand SRL pasireotide binds to SSTR1, 2, 3, and 5, with a binding affinity for SSTR5 that is 39 times higher than octreotide and 106 times higher than lanreotide, as well as a 30-fold higher affinity for SSTR1 compared to octreotide and 19-fold compared to lanreotide (28, 29). Clinically, pasireotide achieves superior suppression of GH and IGF-1 compared with fg-SRLs (30–32).

Currently, the primary drugs used for POMT in acromegaly are fg-SRLs, while the second-generation long-acting SRL, pasireotide, has been studied only in small sample studies from Taiwan and case reports from Japan (33, 34). This suggests that pasireotide requires larger, more comprehensive clinical trials to assess whether it could be a potential treatment option for invasive and fg-SRLs-resistant sparsely granulated adenomas (SGAs).

### 4.2 GH receptor antagonists

Pegvisomant is a pegylated recombinant human GH analogue that treats acromegaly by directly inhibiting the synthesis of IGF-I in the liver. This drug does not act at the pituitary level and therefore does not suppress tumor growth. Initially, clinicians were concerned that reducing IGF-I levels might lead to an increase in the volume of GH-secreting adenomas. However, since tumor growth is typically observed in younger patients with higher GH levels, the 5–10% increase in tumor volume observed during pegvisomant monotherapy is considered more likely to reflect the natural progression of aggressive tumors or a rebound effect after discontinuing prior SRL treatment (35–38). On the other hand, pegvisomant has been demonstrated to effectively control IGF-I levels while improving glucose metabolism (39, 40), obstructive sleep apnea (41), and arrhythmiasin acromegaly patients (42), with neutral or potentially beneficial effects on cardiovascular outcomes (43). Currently, only two cases have been reported involving the preoperative use of pegvisomant (44). As the accessibility and cost of this drug improve in the future, clinicians may consider incorporating it into POMT for acromegaly.

### 4.3 DAs

DAs can bind to dopamine receptor subtype 2 in GH adenomas, reducing the secretion of GH and IGF-1, and inhibiting tumor cell proliferation (45). A small sample study showed that preoperative treatment with cabergoline improved biochemical control in acromegaly patients, with tumor volume changes mainly observed in GH/prolactin co-secreting tumors, and less frequently in tumors that solely secrete GH (46, 47). Although DAs are not an ideal choice for POMT in acromegaly, due to their cost-effectiveness and availability, they may still be explored as part of preoperative combination therapy for patients with incomplete responses to SRLs.

## 5 The clinical treatment benefits of fg-SRLs

Among POMT strategies for acromegaly, fg-SRLs are the most extensively studied, with agents such as octreotide and lanreotide demonstrating potential clinical benefit (**Table 2**). Evidence suggests that a 3–6 month course of fg-SRLs prior to surgery can significantly alleviate common symptoms of acromegaly (48), lower GH and IGF-1 levels (11, 49, 50), and induce a tumor volume reduction of more than 20% in approximately 50% of treatment-naive patients (51–56). Additionally, fg-SRLs have shown beneficial effects on cardiac rhythm and ejection fraction in patients without left ventricular hypertrophy, as well as improvements in lipid profile, blood pressure, and glycemic control (48, 57). However, no significant impact has been observed on immediate postoperative complications or perioperative mortality (51, 58–61). Furthermore, several aspects remain debated, including whether preoperative fg-SRLs influence surgical outcomes by altering tumor consistency, reduce anesthetic risks, or shorten hospitalization (48, 51, 57, 58, 60–62). Further research is needed to clarify their impact on postoperative remission, potential anti-tumor effects, and health economics.

**Table 2:** Comparative summary of studies on POMT for acromegaly.

Study design	Control group	Intervention group	Preoperative intervention & duration	Postoperative follow-up	Remission criteria	Key outcomes	References
Randomized controlled trial	19 patients, transsphenoidal surgery	20 patients, octreotide LAR	Octreotide LAR 20 mg every 28 days for 3 months, intramuscular injection	26.6 ± 4.2 months	2000 consensus	Higher remission at 3 and 6 months; no long−term advantage	(47)
Retrospective cohort	57 patients, transsphenoidal surgery	90 patients, octreotide	Octreotide ≥3 months, mean 221±31 µg/day, subcutaneous injection	51.7 ± 1.4 months	2000 consensus	Slight improvement in remission rate	(48)
Retrospective	—	32 patients, octreotide	Octreotide 300 µg/day, subcutaneous injection	1–3 months	2000 consensus	Overall remission 50%; grade 0: 100%, grade 1: 78%, grade 2: 50%, grade 3: 14%, grade 4: 0%	(49)
Randomized controlled trial	49 patients, transsphenoidal surgery	49 patients, lanreotide	Lanreotide 30 mg every 1–2 weeks for 4 months, intramuscular injection	4 months	2000 consensus	Improved short−term cure rate in newly diagnosed macroadenomas	(50)
Randomized controlled trial	30 patients, transsphenoidal surgery	32 patients, octreotide LAR	Octreotide LAR 20 mg every 28 days for 6 months, intramuscular injection	3 months	2000 consensus	Higher short−term remission in newly diagnosed macroadenomas; no difference in microadenomas or overall cohort	(57)
Randomized controlled trial	25 patients, transsphenoidal surgery	24 patients, lanreotide	Lanreotide 30 mg every 1–2 weeks for 3 months, intramuscular injection	3 months	2000 consensus	Higher short−term remission	(59)
Prospective multicenter	—	104 patients, lanreotide	Lanreotide >1month, subcutaneous injection	Not reported	2000 consensus	Mean volume reduction 25%; 29% achieved >20% shrinkage; high baseline IGF−1, cavernous sinus invasion, and larger volume correlated with lower remission	(61)
Randomized controlled trial	30 patients, transsphenoidal surgery	32 patients, octreotide LAR	Octreotide LAR 20 mg every 28 days for 6 months, intramuscular injection	5 years	2000 consensus	No overall benefit detected	(74)
Retrospective cohort	115 patients, transsphenoidal surgery	96 patients, octreotide LAR or lanreotide	octreotide LAR median total dose 360 mg or lanreotide median total dose 1440 mg; intramuscular injection	≥6 months	2010 consensus	No effect on surgical remission. Preop SRL improved Cormack–Lehane laryngoscopic grade but difficult intubation rates were similar; perioperative complication rates unaffected	(75)
Retrospective cohort	277 patients, transsphenoidal surgery	81 patients, octreotide LAR or lanreotide	Octreotide LAR 20/40 mg monthly or lanreotide 40 mg every 10/14 days, intramuscular injection	≥3 months (range 3–53 months)	2010 consensus	Significantly higher remission in invasive adenomas (Knosp 1–3); improved remission in microadenomas	(77)
Retrospective cohort	46 patients, transsphenoidal surgery	64 patients, octreotide LAR or lanreotide	Octreotide LAR 10/20/30 mg monthly or lanreotide 60/90/120 mg, intramuscular injection	51.4±36.5 months	2010 consensus	Significant improvement in short− and long−term remission in macroadenomas, especially invasive tumors	(82)
Retrospective cohort	62 patients, surgery	38 patients, octreotide LAR or lanreotide	Octreotide LAR 20 mg monthly or lanreotide 40 mg, intramuscular injection	3-6 months	2000 consensus	Improved short− and long−term remission; benefit notable in invasive or macro/giant lesions	(103)

LAR, long-acting release.

### 5.1 Anti-tumor effect

Evidence indicates that preoperative short-acting octreotide can reduce tumor size within 8 weeks (50). In invasive large adenomas, preoperative lanreotide treatment led to a significantly greater tumor volume reduction in patients with short-term remission (60). As pituitary tumor growth reflects the interplay of proliferation, apoptosis, and ischemic or hemorrhagic events, further studies have explored the anti-tumor mechanisms of POMT.

Wasko and colleagues observed that preoperative lanreotide treatment could induce apoptosis in GH-secreting adenoma cells (63). Subsequent studies revealed that patients receiving fg-SRLs treatment showed a significant increase in *in situ* DNA end labeling-positive cells, which correlated positively with the duration of preoperative treatment (63). Similarly, two retrospective studies reported a positive correlation between both the duration and cumulative dose of POMT and the apoptosis index in patients with acromegaly (64, 65). Dagistanli FK et al. reported a significant increase in caspase-3–positive cells in acromegaly tumor tissue, along with a marked decrease in survivin and beclin-1 immunopositivity, and an elevated expression of autophagy-related protein 5 (66). Previous studies have demonstrated that the mesenchymal marker RORC is associated with E-cadherin expression and epithelial-mesenchymal transition (EMT) in acromegaly, both of which are linked to increased tumor size and invasiveness (67–71). Gil et al. investigated the effect of POMT on tumor invasiveness markers and found elevated RORC expression in patients receiving preoperative fg-SRLs (72). Notably, higher RORC levels were observed in those who achieved remission and were significantly correlated with a greater reduction in postoperative IGF-1 levels (72).

Collectively, these studies suggest that fg-SRLs may induce tumor shrinkage in acromegaly by promoting tumor cell apoptosis and inhibiting proliferation. In some patients, the antiproliferative effect of octreotide appears to occur independently of its antisecretory action (50, 53). For invasive large adenomas (Knosp grades 3-4) without visual involvement or pituitary apoplexy, if tumor growth is not progressive after fg-SRLs treatment, extended POMT durations (e.g., 12 months) at maximum tolerated doses may be considered to promote tumor shrinkage, facilitate surgical resection, and improve remission rates.

### 5.2 Improve remission rates

Reducing preoperative IGF-1 and GH levels and shrinking tumor volume with fg-SRLs therapy has long been expected to improve surgical remission rates (11, 73). However, even under the relatively lenient 2000 remission criteria, evidence in GH-secreting pituitary adenomas has been inconsistent, largely due to heterogeneity in study design, sample size, remission definitions, follow-up duration, tumor characteristics, and preoperative treatment regimens (48, 60, 62, 74–76).

Early small-cohort studies may have lacked statistical power to detect significant differences (50, 74, 77), while contemporaneous larger cohorts reported opposite (10). Subsequent retrospective analyses, including a single-center series of 358 patients (78) and multi-center studies (79), confirmed that POMT improved postoperative remission, with case–control data consistently showing benefits for large adenomas (48, 51, 60). Meta-analyses further supported these findings, particularly in patients with suboptimal surgical prognosis (80). To avoid the influence of the lingering effects of preoperative fg-SRLs therapy, these studies delayed postoperative evaluations. However, assessing hormone control 3–4 months post-surgery is generally considered the minimum safe period to eliminate the effects of fg-SRLs. Data collected more than a year post-surgery provides a clearer indication, as it can exclude any residual effects of preoperative fg-SRLs treatment, offering more clinically relevant insights into cure and remission rates (81). Subsequent studies on long-acting fg-SRLs for preoperative treatment have shown less favorable results for long-term remission, with both randomized controlled trials and high-quality meta-analyses demonstrating limited benefits (75, 82). In 2010, revised guidelines introduced more stringent remission criteria, under which long-acting fg-SRLs preoperative treatment was associated with significantly higher long-term remission rates compared to surgery alone (61), and multivariable analysis indicated a significant correlation between POMT and long-term remission (83).

A retrospective study on antitumor characteristics categorized GH adenoma patients into four groups based on tumor size and invasiveness (49). The study found that short-acting octreotide preoperative therapy significantly increased surgical remission rates in the aggressive large adenoma group (49). However, no differences were observed in overall remission rates in the microadenoma group or the non-resectable large adenoma group compared to the control group (49). The results of the subgroup analysis offered valuable insights for future research: tumor size and invasiveness should be considered when selecting and matching patients for POMT. As the Knosp grading system becomes more widely used, researchers have focused on evaluating the benefits of preoperative fg-SRLs therapy on postoperative remission rates in GH adenomas with different Knosp grades. A randomized controlled trial found that acromegaly patients with Hardy-Knosp grades III-IV benefited significantly from POMT compared to those with Hardy-Knosp grade V (48). Cohort studies demonstrated that POMT significantly improved remission rates for invasive large adenomas with Knosp grades 1-3, increasing from 37.3% to 56.4% (78). However, it did not enhance remission rates for microadenomas or large adenomas with Knosp grades 0-2 (84). Additionally, POMT showed no benefit in postoperative remission for invasive large adenomas with Knosp grade 4 (50, 78).

In terms of drug selection and treatment duration, early studies used short-acting octreotide administered three times daily, with daily doses ranging from 100 to 1500 µg and treatment durations varying from 2 weeks to 39 months (10, 49, 50, 77). Tumor reduction in pituitary GH adenomas reached near its maximum after 3–4 months of high-dose short-acting octreotide therapy (50). This finding provided a reference for the duration of POMT, and subsequent randomized controlled trials typically designed treatment durations of 3–6 months (48, 51, 60, 75). With the development of long-acting fg-SRLs, the treatment regimen for POMT has progressed from octreotide microspheres and lanreotide acetate to the current formulations of octreotide microspheres and lanreotide acetate long-acting injections, with dosing schedules of 20–30 mg every 28 days and 60–120 mg, respectively (83, 85, 86). A recent study further revealed that preoperative treatment with ≥30 mg of octreotide or ≥90 mg of lanreotide acetate long-acting injection significantly improved long-term surgical remission rates compared to lower-dose treatments or untreated patients (61). A multicenter study in Poland administered 6–12 months of preoperative octreotide microsphere therapy for large adenomas to achieve maximal reduction in GH and IGF-1 concentrations (85). During the treatment period, 49.1% of large adenomas experienced a ≥20% reduction in volume, and GH and IGF-1 levels decreased by 49% and 40%, respectively (85). The 12-month POMT treatment was found to be safe and well-tolerated (85). However, another study showed no superior results in tumor volume reduction and endocrine control compared to a concurrent large-scale study conducted in China, which used 3 months of preoperative octreotide microsphere therapy for GH adenomas (86). Unfortunately, neither of these studies directly compared the treatment duration of octreotide microsphere preoperative therapy.

For invasive large adenomas with Knosp grade 4, multimodal therapy remains standard. In contrast, Knosp grades 1–3 warrant careful POMT consideration, as high-dose SRLs may enhance SSTR5 binding, upregulate SSTR2, and promote greater tumor shrinkage (80). Given that the most pronounced volumetric response occurs within the first year, large-scale prospective trials are needed to define the optimal dosing and duration for maximizing surgical outcomes (54).

### 5.3 Reducing medical costs

Colao et al. explored the cost-effectiveness of short-acting octreotide POMT, finding it offers a better cost-benefit ratio than surgery alone (57). Two subsequent studies confirmed that, in centers with suboptimal surgical outcomes, preoperative fg-SRL treatment not only improved surgical results but also provided long-term cost savings (87, 88). One study focused solely on pharmacoeconomic costs (87), while the other employed a dynamic Markov model to comprehensively assess costs, including diagnostics, treatment (surgery, medication, or radiotherapy), complications, and disease monitoring (88). However, an opposing study, with a shorter evaluation period and smaller sample size, found no significant cost-effectiveness advantage for POMT (89).

In summary, the use of fg-SRLs for preoperative treatment in acromegaly has gained continued attention and influenced clinical practices in several countries (90). While debates persist, the benefits of POMT are becoming clearer, particularly as study sample sizes grow, study designs improve, remission criteria and follow-up periods are standardized, and participant characteristics are refined. This trend is especially evident in patients who are unlikely to achieve remission post-surgery or have surgical contraindications, as well as those treated in centers with suboptimal surgical outcomes.

### 5.4 Preoperative SRLs in patients with airway/anesthetic risk

Patients with acromegaly frequently have airway abnormalities and obstructive sleep apnea (OSA), which increase the risk of difficult laryngoscopy and traumatic intubation (41). In selected high-risk patients (e.g., marked oropharyngeal soft-tissue thickening, macroglossia, or severe OSA), short-term preoperative SRLs therapy may be considered to reduce soft-tissue edema and optimize peri-anesthetic conditions (41). Notably, in a comparative series, SRLs pretreatment improved the Cormack–Lehane laryngoscopic grade, yet the overall rate of difficult intubation did not differ from surgery-alone controls, indicating that airway benefits may not translate into fewer difficult intubations at the cohort level and should be individualized to patients at highest risk (76).

## 6 Identifying candidates for preoperative fg-SRLs therapy

Many studies have investigated clinical and imaging predictors of postoperative biochemical remission to assist in preoperative decision-making. For patients assessed to have a low chance of surgical cure based on clinical parameters such as age, sex, preoperative biochemical markers, tumor size, CSI, and bilateral internal carotid artery distance (91–94), multi-modal treatments, including POMT, should be considered, especially for those treated at surgical centers with less experience (95–97).

However, not all patients with low expected TSS remission rates are suitable for POMT. Evidence shows that up to 45% of acromegaly patients have poor or no response to fg-SRLs, potentially due to varying expression of SSTR subtypes in tumors (98, 99). While SSTR phenotype and tissue patterns predict SRL treatment efficacy, they do not directly guide preoperative decisions (100). Therefore, clinical pre-stratification methods are needed to identify patients most likely to benefit from fg-SRLs-based POMT, enabling personalized and precise treatment (**Table 3**).

**Table 3:** Preoperative assessment strategies for predicting responsiveness to fg-SRLs in patients with acromegaly.

Preoperative assessment modality	Predictive roles	Advantages	Limitations
Basic clinical and conventional imaging factors (e.g., age, sex, IGF-1, tumor size, Knosp grade)	Reflect overall disease burden, metabolic activity, and invasiveness; influence pharmacological responsiveness	Readily available; allow stratification using multivariate models	Non-specific; cannot fully capture tumor heterogeneity; thresholds may vary across cohorts
Advanced imaging techniques (e.g., T2-weighted MRI signal, radiomics features)	T2 hypointensity is associated with densely granulated tumors and SSTR2A overexpression; radiomics captures intratumoral heterogeneity	Non-invasive; can predict SRL responsiveness and tumor subtype; emerging as personalized imaging biomarkers	T2 signal interpretation lacks standardization; radiomics requires specialized software and prospective validation
Functional testing (e.g., short-acting octreotide suppression test)	Evaluate short-term GH/IGF-1 reduction after single-dose octreotide to predict long-acting SRL response	Practical; may forecast biochemical response to fg-SRLs in sensitive patients	Protocols vary (dose, timing, evaluation criteria); inconclusive for predicting tumor shrinkage or surgical remission
Molecular biomarkers (e.g., SSTR2, E-cadherin, SNAI1, RORC)	Reflect receptor expression and signaling pathways related to SRL resistance (e.g., EMT, β-arrestins)	High specificity; may enable individualized POMT selection via tissue or liquid biopsy in the future	Still investigational; no clinical consensus; requires NGS or immunohistochemistry; limited preoperative accessibility

fg-SRLs, first-generation somatostatin receptor ligands; GH, growth hormone; IGF-1, insulin-like growth factor 1; BMI, body mass index; Knosp grade, grading system for cavernous sinus invasion by pituitary adenomas; MRI, magnetic resonance imaging; SSTR2A, somatostatin receptor subtype 2A; EMT, epithelial–mesenchymal transition; NGS, next-generation sequencing; POMT, pre-surgical medical therapy.

### 6.1 Clinical baseline information predictors for preoperative fg-SRLs therapy

Certain preoperative clinical information can predict the response to fg-SRLs treatment. Younger male patients generally exhibit poorer responses to SRLs (101), while factors such as lower GH levels at diagnosis, smaller tumor size, and the absence of cavernous sinus invasion are closely linked to better hormonal responses to SRL treatment (56). A maximum tumor diameter of 2 cm is a strong predictor of both biochemical response and tumor shrinkage, and is correlated with the proportion of SG adenomas and Ki-67 expression (63). A meta-analysis using multivariate regression models on 622 patients from two European cohorts found that baseline IGF-1 was the best predictor of biochemical response to fg-SRLs, followed by body mass index (BMI) (102). Furthermore, combining diagnostic age, previous surgery, and tumor size helped identify non-responders (102).

Some of these predictive factors also serve as prognostic indicators for surgical outcomes after preoperative fg-SRLs treatment. A *post-hoc* analysis of the PRIMARYS study found that older age, female sex, and lower baseline IGF-I levels were associated with a higher likelihood of long-term biochemical control following long-acting SRL injection (103). Tumor invasiveness is also significantly related to both short-term and long-term remission benefits from POMT, and POMT is recommended as first-line treatment for patients with cavernous sinus invasion (51, 104, 105).

### 6.2 Innovative imaging technology for preoperative fg-SRLs therapy

With the widespread use of magnetic resonance imaging (MRI) in the diagnostic imaging of pituitary tumors, researchers have found that tumors with low T2-weighted signal on MRI typically respond better to SRLs than those with equal or high signal (106–108). This is explained by the correlation between low T2 signal and high expression of SSTR2A on the cell membrane of densely granulated (DG) tumors (109). The expression of SSTR2A is higher in DG tumors than in sparsely granulated (SG) tumors, and patients with SSTR2A-positive tumors show a better response to fg-SRLs, making DG tumor patients respond significantly better to this treatment (100). In recent years, researchers have further explored quantitative T2-weighted MRI techniques, such as T2 homogeneity ratios and relative signal intensities, to predict tumor volume reduction >20%, combining these methods with qualitative T2-weighted MRI to better predict histological patterns (110). New radiomics methods, such as quantitative texture analysis, have also been applied to assess the response to fg-SRLs (111). In terms of functional imaging, initial explorations of 111Indium-pentetreotide scintigraphy ± SPECT (octreoscan) and 68Gallium-DOTATE PET/CT have not demonstrated clear clinical effectiveness in predicting SRL response (112, 113).

### 6.3 Functional testing for preoperative fg-SRLs therapy

Early studies on the use of short-acting octreotide in preoperative treatment aimed to assess the effectiveness of fg-SRLs therapy through functional tests. Over the following decades, a series of investigations were conducted, though methodological variations were present. Most studies involved a single subcutaneous injection of 100 µg of octreotide, with some using alternative dosages or multiple daily injections. The timing of blood sampling after administration was not standardized, and evaluation criteria varied, leading to differing conclusions.

Several studies have suggested that the octreotide suppression test is closely correlated with a reduction in preoperative serum GH levels and can predict the biochemical response after octreotide treatment (50, 114, 115). This appears to be a key factor for achieving postoperative biochemical control in POMT patients. However, the effect of the octreotide suppression test on tumor volume and postoperative remission rates remains debated. Notably, a positive response to either octreotide or bromocriptine suppression tests was not associated with tumor reduction (50, 53). However, a positive response to both tests was significantly correlated with tumor shrinkage (50). The octreotide suppression test demonstrated no predictive value in relation to postoperative remission. Nevertheless, Annamalai AK et al. found that GH reduction after a 100µg subcutaneous octreotide injection correlated with changes in GH, IGF-1, and tumor volume after 6 months of lanreotide therapy, and was predictive of short-term postoperative remission (116). Despite lacking specific sampling time points for GH nadir, responders showed a GH reduction of over 69%, higher than the criteria used in other studies (116). Even patients categorized as non-responders showed GH reductions of 29.2% and 30.1%, respectively (116). These findings suggest the need for further exploration of the octreotide suppression test evaluation criteria.

Studies examining the effects of various drugs have demonstrated that the octreotide suppression test is only capable of predicting the long-term efficacy of short-acting octreotide and offers no predictive value for lanreotide acetate (114). Within the first six months of treatment, short-acting octreotide leads to a more rapid and significant reduction in IGF-I and GH compared to lanreotide acetate. Therefore, short-acting octreotide is recommended for preoperative short-term treatment, while lanreotide acetate is preferred for long-term postoperative treatment (114). However, several meta-analyses on preoperative SRL therapy have shown that lanreotide is as effective as octreotide in improving short-term postoperative remission rates (116, 117). These studies included short-acting octreotide, lanreotide acetate, and octreotide microspheres. With advances in acromegaly treatment, octreotide microspheres and long-acting lanreotide acetate are increasingly used in POMT, providing valuable insights for clinical practice. The findings from these meta-analyses may provide further guidance for clinical practice.

Additionally, evidence suggests that GH reduction after the octreotide suppression test is significantly greater in Knosp grade 3–4 GH adenomas compared to grade 0-2 (50). Whether the same dosing methods and evaluation criteria should be applied to tumors with different levels of invasiveness, along with the integration of new imaging techniques and functional test results to predict the effects of preoperative SRLs therapy, remains a key area for future research.

### 6.4 Molecular biomarkers for preoperative fg-SRLs therapy

In addition to SSTR2 and the granule pattern, several molecular biomarkers can predict the response to SRLs. Among them, E-cadherin has garnered significant attention, as its loss is a key event in EMT and is associated with a lack of SRLs response in tumors (71). The mesenchymal marker Snail family transcriptional repressor 1 (SNAI1), a direct inhibitor of E-cadherin, also plays a crucial role as a transcription factor in EMT regulation (118). Gil et al. found that adenomas with extrapituitary growth expressed higher levels of SNAI1 and were the first to report a relationship between SNAI1 expression and poor SRLs response (72). Other molecular biomarkers, including RORC, β-arrestins expression, aryl hydrocarbon receptor interacting protein, and Survivin, have also been identified as potential preoperative predictors of fg-SRLs response (118–120). Further research is expected to enable preoperative assessment of these biomarkers using methods like Next-Generation Sequencing (NGS) from blood samples to predict treatment response.

**Figure 1:**
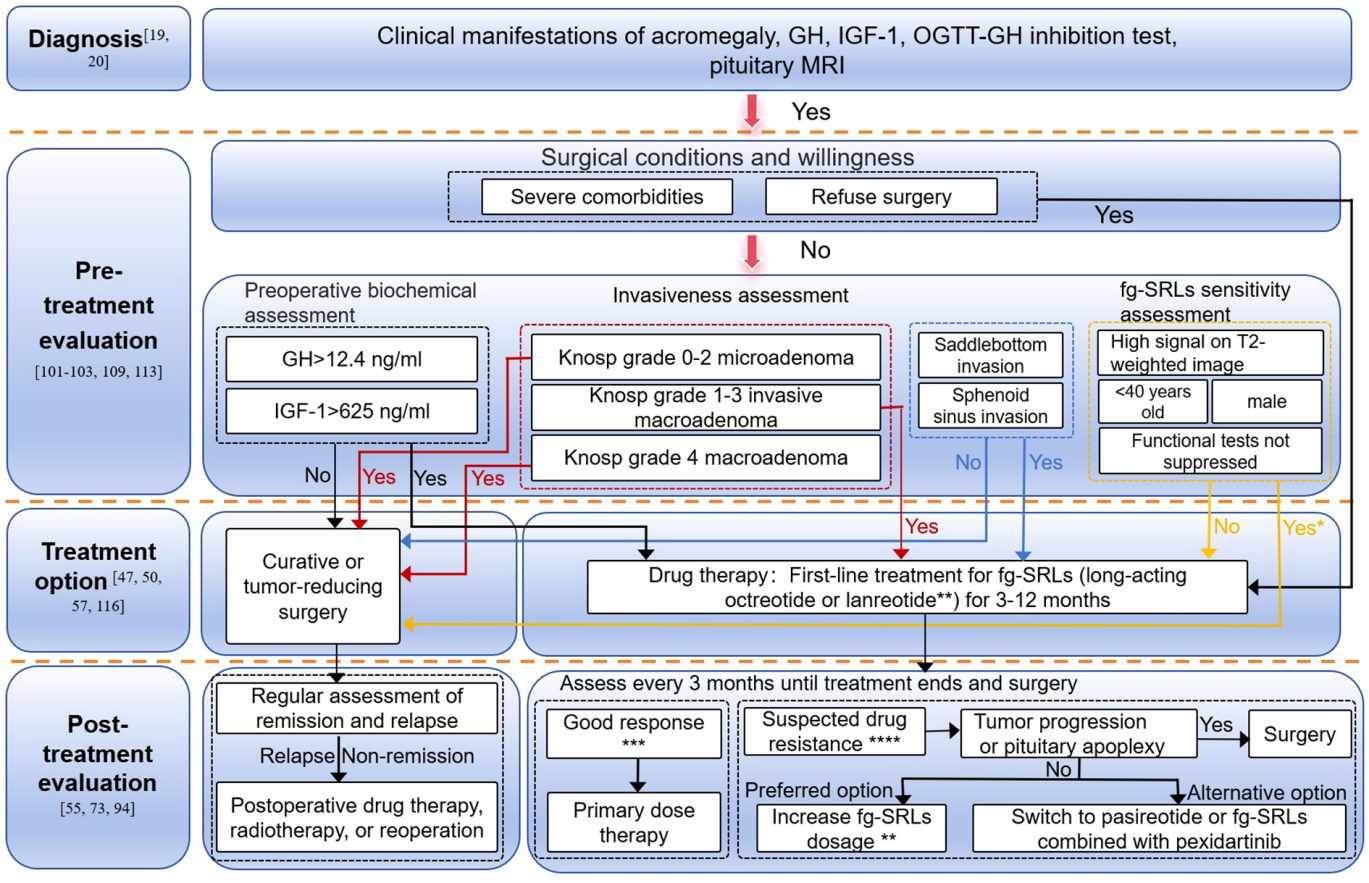
POMT workflow for acromegaly. *In surgical centers with experienced surgeons or those utilizing technologies such as neuronavigation, patients with suspected drug resistance based on fg-SRLs sensitivity assessments should ideally be treated surgically. Otherwise, POMT can be attempted, with prompt referral to an experienced surgical center for surgery if necessary. **Long-acting octreotide 20–30 mg every 28 days or long-acting lanreotide 60–120 mg every 28 days, starting with a low dose and adjusting dosing intervals and amounts based on evaluation results; ***Good response: Biochemical response (normalization of IGF-1 or ≥rF- reduction) and tumor size response (tumor shrinkage >20%); ****Drug resistance: Normalization of hormonal deficiency, tumor enlargement, or tumor shrinkage of less than 20% of baseline volume.

Based on these findings, it is evident that previous studies on whether POMT improves the ultimate outcomes of acromegaly have not assessed and differentiated SRL responders and non-responders before administration. Further comparisons are needed to evaluate the impact of preoperative integrated assessment and tailored SRL treatment on improving surgical remission rates in responsive patients. In this context, our study proposes a POMT flowchart to assist in making individualized treatment decisions (**Figure 1**). Although several molecular biomarkers (e.g., β-arrestin, E-cadherin) have been explored to differentiate responders from non-responders, robust and validated assays suitable for preoperative use are not yet available. Future work should develop externally validate preoperative biomarker panels and predictive models, and conduct multicenter, prospective comparative studies.

## 7 Clinical potential of new SRLs formulations for POMT

To improve remission rates in treatment-resistant patients and adherence in those intolerant to current therapies, novel formulations of existing SRLs are under development. As injectable SRLs remain the cornerstone of POMT, evaluating their new delivery forms is essential. Oral octreotide, a new formulation that combines octreotide with permeability enhancer technology, addresses issues like injection site pain, induration, and the inconvenience of hospital visits (121). It can improve patient adherence, enhance quality of life during treatment, and allow more flexible dosage adjustments (122). However, there are no reports yet on the tumor-shrinking effects of oral octreotide on pituitary tumors. Considering that improving biochemical control and reducing tumor volume are the main goals of preoperative SRLs therapy, further studies on its role in POMT may be required once data on tumor-shrinking effects are available. Additionally, the development of water-soluble co-solvents, FluidCrystal technology, needle-free solid dosage injectors, and intranasal formulations could improve patient adherence and the possibility of self-administration. These drugs are still in phase I-III clinical trials, and POMT-related studies will need to wait for data on their effects on biochemical control and tumor shrinkage before they can be considered for further development.

## 8 Conclusions and future perspectives

In conclusion, the treatment of acromegaly remains challenging, requiring multimodal therapies, including POMT, to improve patient prognosis. fg-SRLs, extensively studied for preoperative treatment, have shown benefits despite some controversy. The effects of these drugs on postoperative remission rates, anti-tumor activity, molecular biomarkers, and health economics continue to attract attention, with increasingly clear results, with clearer results emerging. Models incorporating various clinical factors and functional tests, especially the exploration of new imaging techniques and molecular biomarkers, may help identify patients who would benefit from POMT among those with suboptimal TSS outcomes. Patients with acromegaly frequently present with airway abnormalities and obstructive sleep apnea (OSA), contributing to difficult laryngoscopy/intubation. Guidelines suggest that preoperative SRLs may be considered in patients with severe pharyngeal thickening or OSA to reduce soft-tissue edema, improve OSA, and potentially lower intubation-related complications. Nevertheless, in a comparative series, SRLs pretreatment improved the Cormack–Lehane grade but did not reduce the overall rate of difficult intubation, underscoring that SRL use for airway optimization should be individualized to those at highest anesthetic risk. fg-SRLs remain the cornerstone of preoperative treatment, with most clinical studies currently focused on these agents. However, the routine use of growth hormone receptor antagonists (e.g., pegvisomant) and dopamine agonists (e.g., cabergoline) in POMT still lacks sufficient clinical evidence. While these therapies may hold potential in select cases, further research is necessary to establish their role in preoperative treatment.

Further research into whether POMT for SRL-sensitive patients improves surgical remission rates is needed. Furthermore, new formulations of existing SRLs and potential molecular targets may offer new insights and directions for POMT development in acromegaly.
